# Supplementary material for: Effect of Upper Limb Motor Rehabilitation on Cognition in Parkinson’s Disease: An Observational Study
Source: Brain Sci. 2022 Dec 8;12(12):1684. doi: 10.3390/brainsci12121684 (PMC9775162; doi:10.3390/brainsci12121684)

ARMSHAKE DEVICE EXERCISE PROGRAM

DR. OLIVER KROMAT

move it
SYSTEME FÜR BEWEGUNG

WARM UP

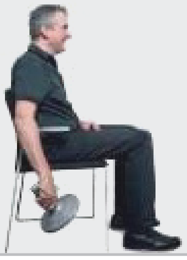

Comfortable sitting position: let the right arm hang down naturally (slight angle in the elbow joint). Keep your hand tight on the tool. Note: try to relax your arm and shoulder during the exercise and try to sit upright as much as possible. Change the arm and repeat the exercise.
Intensity: medium-low
Duration: 30-60 sec

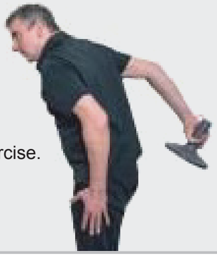

Standing. Relaxed position, feet wide: put your right arm behind your back and keep the elbow joint slightly flexed. Keep your hand tight on the tool. Place your free hand attached to the corresponding side of the body. Note: try to relax your arm during the exercise and stay upright as much as possible. Change arm and repeat the exercise.
Intensity: medium-low
Duration: 30-60 sec

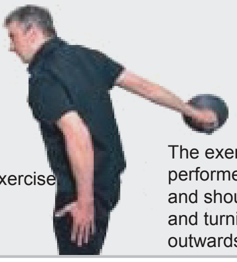

The exercise should be performed with the elbow and shoulder extended and turning the thumb outwards.

MOBILIZATION FOR THE ARTICULATION OF THE HAND (WRIST) FROM THE SEAT

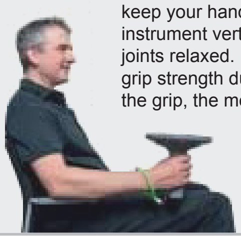

Comfortable sitting position on a chair with backrest: place your forearm on the armrest of the chair and keep your hand close to the instrument and the instrument vertically. Keep the upper limb joints relaxed. Note: sit upright and change the grip strength during the exercise; the stronger the grip, the more the arm swings.
Change the arm and repeat the exercise.
Intensity: medium-low
Duration: 30-60 sec

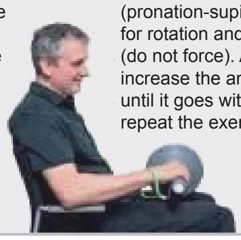

Starting position: as before. During the exercise, start by turning the hand inwards and outwards (pronation-supination), choose a moderate speed for rotation and rotate the wrist as far as you can (do not force). After 3/5 workouts, you can try to increase the angle of rotation of the hand but only until it goes without pain. Change the arm and repeat the exercise.
Intensity: medium-low
Duration: 30-60 sec

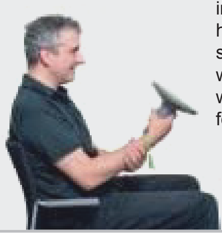

If the arm alone is not able to support the instrument, you can help yourself by supporting the arm with the other arm, with a grip on the forearm.

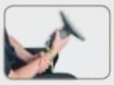

MOBILIZATION FOR THE HAND AND SHOULDER, FROM THE SEAT

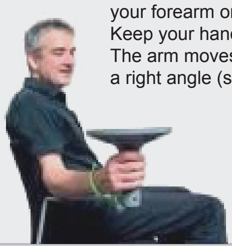

Comfortable sitting position:first place your forearm on the armrest of the chair. Keep your hand tight and the tool vertical. The arm moves away from the body at a right angle (shoulder external rotation).

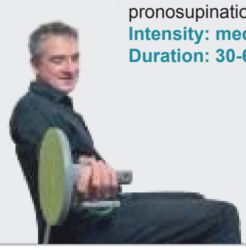

From this position perform pronosupination movements.
Intensity: medium-low
Duration: 30-60 sec

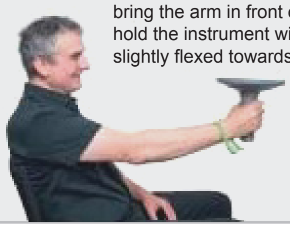

We conclude this sequence of exercises with the passage to the next. To do this bring the arm in front of the body and hold the instrument with the elbow joint slightly flexed towards the chest.
Repeat the last three exercises with the other hand.

MOBILIZATION AND STABILIZATION FOR ARM AND SHOULDER

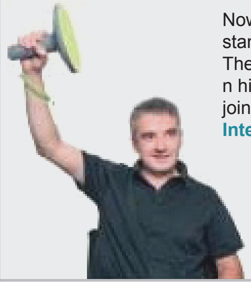

Now in a standing position, stand with your legs apart. Then he puts on the tool, holding it n his hand, above the head - the elbow joint remains slightly flexed.
Intensity: high

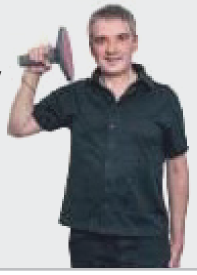

Then move the instrument with the flat side towards the ear and remain in this position.
Intensity: high
Duration: 10-20 sec.

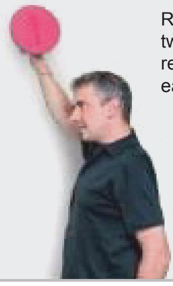

Repeat the last two exercises two to three times and slightly reduce the frequency with each repetition

SENSOMOTOR FUNCTION FOR HANDS ANDFOREARMS

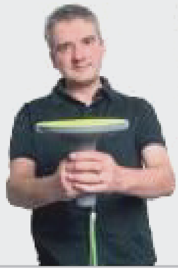

This exercise is performed standing up again. To sync both hands now, hold the instrument with both hands firmly closed (flat side upwards). Note: also in this case, vary the grip force to control the strength of the wave in the arm and in the body.
Intensity: medium-high
Duration: 20-30 sec.

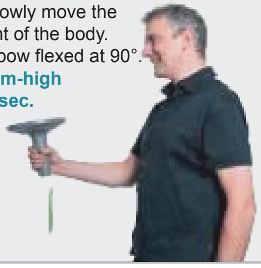

Release one hand from the instrument and slowly move the other hand in front of the body. Keep arm with elbow flexed at 90°.
Intensity: medium-high
Duration: 10-20 sec.

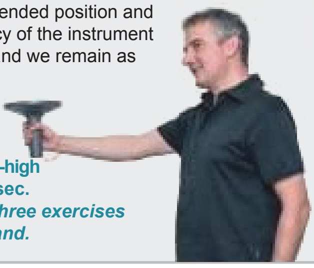

At the end of the intensive phase we move the arm to the extended position and raise the frequency of the instrument to the maximum and we remain as long as possible, but not more than a minute.
Intensity: medium-high
Duration: 10-60 sec.
Repeat the last three exercises with the other hand.

COOL DOWN

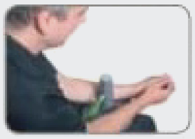

At the end of the workout we want to relax. For this we sit on the chair, we put the medium-low frequency instrument with the flat part resting on the legs and let's put arms resting on the instrument (and hands joined). Note: close your eyes and make sure you breathe deeply and calmly.
Intensity: medium-low
Duration: 30-60 sec.

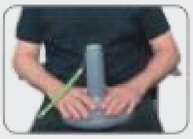

Now place your hands on the instrument and rest your elbows on the armrests of the chair.
Intensity: medium-low
Duration: 30-60 sec.

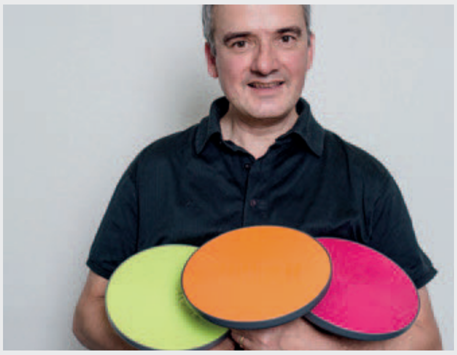

Supplement: Supplementary file 1 [file brainsci-12-01684-s001.zip › File S1 Exercises program Armshake.pdf]
