# Supplementary figures and images for: Effect of Upper Limb Motor Rehabilitation on Cognition in Parkinson’s Disease: An Observational Study
Source: Brain Sci. 2022 Dec 8;12(12):1684. doi: 10.3390/brainsci12121684 (PMC9775162; doi:10.3390/brainsci12121684)

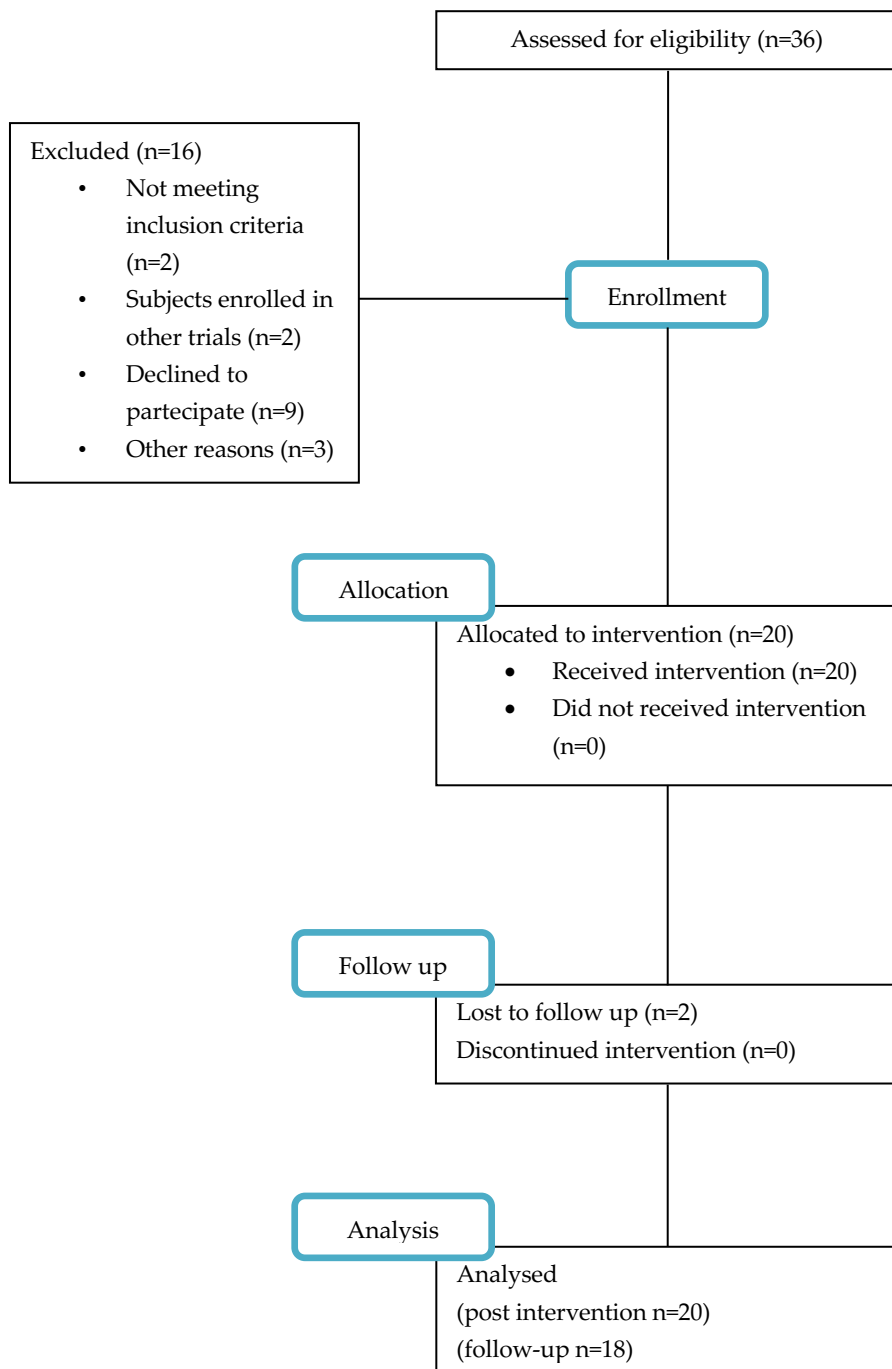

Supplement: Supplementary file 1 [file brainsci-12-01684-s001.zip › Figure S1 flowchart_armshake_cog.pdf]
